# Predictors of HIV serostatus disclosure to partners among HIV-positive pregnant women in Morogoro, Tanzania

**DOI:** 10.1186/1471-2458-13-433

**Published:** 2013-05-03

**Authors:** Elizabeth S Kiula, Damian J Damian, Sia E Msuya

**Affiliations:** 1 Morogoro School of Public Health Nursing, Po Box 1060, Morogoro, Tanzania; 2 Kilimanjaro Christian Medical University College, Po Box 2240, Moshi, Tanzania; 3 Kilimanjaro Christian Medical Centre, Po Box 3010, Moshi, Tanzania

**Keywords:** HIV, Disclosure, Pregnant women, PMTCT, Male partner, Tanzania

## Abstract

**Background:**

Prevention of mother to child transmission of HIV (PMTCT) has been scaled, to more than 90% of health facilities in Tanzania. Disclosure of HIV results to partners and their participation is encouraged in the program. This study aimed to determine the prevalence, patterns and predictors of HIV sero-status disclosure to partners among HIV positive pregnant women in Morogoro municipality, Tanzania.

**Methods:**

A cross sectional study was conducted in March to May 2010 among HIV-positive pregnant women who were attending for routine antenatal care in primary health care facilities of the municipality and had been tested for HIV at least one month prior to the study. Questionnaires were used to collect information on possible predictors of HIV disclosure to partners.

**Results:**

A total of 250 HIV-positive pregnant women were enrolled. Forty one percent (102) had disclosed their HIV sero-status to their partners. HIV-disclosure to partners was more likely among pregnant women who were < 25 years old [Adjusted odds ratio (AOR) = 2.2; 95% CI: 1.2–4.1], who knew their HIV status before the current pregnancy [AOR = 3.7; 95% CI: 1.7–8.3], and discussed with their partner before testing [AOR = 6.9; 95% CI: 2.4–20.1]. Dependency on the partner for food/rent/school fees, led to lower odds of disclosure to partners [AOR = 0.4; 95% CI: 0.1–0.7]. Nine out of ten women reported to have been counseled on importance of disclosure and partner participation.

**Conclusions:**

Six in ten HIV positive pregnant women in this setting had not disclosed their results of the HIV test to their partners. Empowering pregnant women to have an individualized HIV-disclosure plan, strengthening of the HIV provider initiated counseling and testing and addressing economic development, may be some of the strategies in improving HIV disclosure and partner involvement in this setting.

## Background

HIV/AIDS and mother-to-child transmission of HIV (MTCT) are still major public health problems in sub Saharan Africa (SSA). SSA has an estimated 68% of the 33 million people living with HIV (PLWHIV) globally, nearly 90% of pregnant women living with HIV and more than 90% of HIV infections among children < 15 years old, globally [1-3]. Strategies to prevent new infections among the uninfected and prevention of onward vertical or sexual transmission from HIV infected are therefore vital if the MDG 6 of halting and reversing the trend of new HIV infections is to be met by SSA countries in 2015.

Prevention of mother-to-child transmission of HIV (PMTCT) programs are now available in many low and middle income countries [4]. Disclosure or sharing of HIV status with ones sexual partner is encouraged and is an integral practice both in VCT and PMTCT programs [2-5], though it is complex and challenging [6-11]. The Policy in PMTCT programs advocates that all pregnant women, negative and positive, should be empowered to disclose their HIV status to their sexual partners through counselling [2,4,5]. Among HIV-positive pregnant women, studies has shown that disclosure of HIV status to partners led to increased - acceptance, use and adherence to maternal and infant ARVs, improved adherence to infant feeding method selected, increased use of cotrimoxazole prophylaxis, decreased mortality and increased survival and follow up among HIV exposed infants [6,12-16].

HIV testing of pregnant women should not only be the entry point to PMTCT of HIV, but also considered as an opportunity for prevention of sexual transmission of HIV. Studies have shown that, a substantial proportion of HIV infected individuals are in stable partnership with HIV uninfected partners in SSA. The prevalence of HIV-serodiscordance ranges from 8 -14% in eastern Africa and 14-27% in southern Africa [17,18]. A large proportion of new HIV infections in SSA occur among discordant couples, and disclosure may be one of the key strategies in reducing HIV transmission [9,17-20]. Studies have shown that disclosure to partners increased couple dialogue, communication and engagement in preventive behavior, increased male partner testing, increased adherence of ARV treatment regimens and improved use of condoms and other forms of contraception [9,12,14,19,21]. Negative events were fewer than feared earlier when women disclose their HIV status to their partner [11,12,14,19,22].

The prevalence of HIV sero-status disclosure to sexual partners has shown to differ between populations. Prevalence of disclosure among women attending VCT ranged between 69% - 86% [7,11,23], 80 -86% among women attending ARV care and treatment clinics [10,23,24], and was lowest among pregnant women 16.7% - 46% [8,9,14,19,21,25]. Disclosure of HIV status is influenced by a number of factors. It depends on whether the partnership is regular rather than casual or an unfamiliar relationship [23,24,26], a polygamous marriage [14,21], whether the person being disclosed to has a known positive status [10,23,24], the number of lifetime sex partners [25] and fears of abandonment, discrimination, violence and accusation of infidelity [8,9,11,23,25].

In Tanzania the national PMTCT programme was introduced in 2004, and by the end of 2011, 91% of the health facilities with reproductive and child health (RCH) services were offering PMTCT service [5]. While systematic data is available on the proportion of pregnant women who have ever tested for HIV, information on the pattern of HIV disclosure to partners is not available, and thus limited in Tanzania [5,27]. Information on HIV disclosure patterns among pregnant women which is available was collected before the introduction and scale up of the national program in 2004 [9,25]. The rates and reasons affecting disclosure may have changed over time, and need to be documented so that interventions to facilitate disclosure between partners are adapted accordingly. This study aimed to determine the prevalence of, and predictors of HIV sero-status disclosure to partners among pregnant women in Morogoro Municipality located in south eastern Tanzania.

## Methods

### Study design and site

This cross sectional study was conducted between March and May 2010 among HIV positive pregnant women in Morogoro Municipality, Tanzania. Morogoro Municipality is one of the six districts of Morogoro region situated in south eastern Tanzania, about 190 kilometers west of Dar es Salaam. The district has an approximate population of 294,467, with the main economic activities for about 90% of the population being small scale farming, keeping livestock, small scale trade and working in the textile industry [28].

Administratively, the district has one division, 19 wards and 274 hamlets. At the time of the study, the municipality had no district hospital, but it had four government health centres and 12 dispensaries. All of the 16 government health facilities offered RCH services including routine antenatal care, child growth monitoring, vaccination, family planning, HIV counseling and testing for pregnant women and offering antiretroviral prophylaxis and other necessary care for HIV positive women and their infants.

Attendance for RCH services is high; 98% of women attend for antenatal care, 78% of children have been fully vaccinated and 61% deliver with skilled birth attendants, compared to the national level of 96%, 75% and 50% respectively [27]. The HIV prevalence among adults aged 15–49 years was estimated to be 4.2%; with women having a higher prevalence (6%) than men (2%) [29]. Ninety one percent of women and men know where to get tested for HIV, but only 53% and 37% of women and men of reproductive age respectively, have ever been tested for HIV in Morogoro.

### Population and enrolment procedures

Simple random sampling was used to select six government health facilities out of 16 to participate in the study. Three health centres and three dispensaries participated. All the HIV positive pregnant women who were attending the antenatal clinics for routine care during the study period were eligible to participate. We limited the inclusion to those HIV positive pregnant women who had undergone counselling and received their HIV test results at least one month prior to the study.

All the HIV positive women meeting inclusion criteria were invited to participate. After obtaining informed consent, two trained researchers administered a questionnaire during face to face interviews (a questionnaire can be accessed at Additional file 1). The collected information included; socio-demographic information (age, education, marital status, occupation), socio-economic and household information (income per month, assets, ownership of house, and payment for food, fees or rent), sexual and reproductive health information (parity, birth spacing, use of condoms), knowledge of PMTCT in general (transmission and prevention), disclosure to partners and other relatives and, last perceived benefits and challenges of disclosure of HIV status. Information on sexual partners socio-demographic, reproductive and previous history on HIV- discussion and testing was also collected.

### Statistical analysis

Descriptive statistics was used to summarize the data. Bivariate analysis was used to examine associations between dependent variable (HIV disclosure to partner) and explanatory variables by using *χ*^2^ test. Variables found to be significant at 5% level in the bivariate analyses were entered in the logistic regression model, to get independent predictors of HIV serostatus disclosure to the partner. Data were analyzed using SPSS statistical software, version 16.0 (SPSS, Chicago, IL, USA).

### Ethical approval

Ethical clearance was obtained from the Ethical Committee of Kilimanjaro Christian Medical University College, Tumaini University. Permission to conduct the research was also obtained from the Medical Officer in charge of Morogoro Municipality and the heads of the respective clinics where the study was conducted. Individual written informed consent was obtained from every HIV positive pregnant woman who agreed to participate in the study.

## Results

### Socio-demographic characteristics of respondents

A total of 250 HIV positive pregnant women out of 265 participated in the study (response rate 94%). Their age ranged from 17 to 41 years with a mean (±SD) of 27 (±5.3) years. A majority of the study participants were married/cohabiting (92.0%), had primary education (69.0%), were unemployed (78.8%), dependent on partner for rent/food or school fees (83%), had ≤ 2 children (79%), with age of the last born being > 2 years for 98% of the women. In Tables 1, 2 and 3, the basic socio-demographic, economic, and reproductive descriptions of the participants are depicted.

**Table 1:** The association between socio-demographic characteristics and HIV-serostatus disclosure to partner/spouse (n = 250)

**Characteristics**	**Total n (%)**	**Disclosed (n = 102) n (%)**	**OR (95% CI)**	**P-value**
***Age (years):***				
≥ 25 years	158 (63.2)	56 (35.4)		
< 25 years	92 (36.8)	46 (50.0)	1.8 (1.1-3.1)	0.024
***Religion:***				
Christian	135 (54.0)	50 (37.0)		
Moslem	115 (46.0)	52 (45.2)	1.4 (0.8-2.3)	0.190
***Education level:***				
None/primary level	193 (77.2)	70 (36.3)		
Secondary and above	57 (22.8)	32 (56.1)	2.2 (1.2-4.1)	0.007
***Marital status:***				
Married/cohabiting	230 (92.0)	90 (39.1)		
Single/divorced/widowed	20 (8.0)	12 (60.9)	2.3 (0.9-5.9)	0.007
***Currently living with partner/spouse:***				
Yes	208 (83.2)	82 (39.4)		
No	42 (16.8)	20 (47.6)	1.4 (0.7-2.7)	0.324
***Duration of living together for married/cohabiting (n = 230):***				
> 2	163 (70.9)	57 (35.0)		
≤ 2	67 (29.1)	33 (49.3)	1.81 (1.0-3.2)	0.041
***Partner’s age (years):***				
≤ 35 years	129 (51.6)	51 (39.5)		
> 35 years	121 (48.4)	51 (42.1)	1.1 (0.7-1.8)	0.674
***Partner’s education level:***				
None/primary	145 (58.0)	49 (33.8)		
Secondary and above	105 (42.0)	53 (50.5)	2.0 (1.2-3.3)	0.008
***Partner’s occupation status:***				
Not employed	161 (64.4)	64 (39.8)		
Employed	89 (35.6)	38 (42.7)	1.1 (0.7-1.9)	0.650
***Age difference between partners (years)***				
≤ 10 years	209 (83.6)	77 (36.8)		
> 10 years	41 (16.4)	25 (61.0)	2.7 (1.3-5.3)	0.004

**Table 2:** Socio-economic factors influencing self disclosure of HIV- serostatus to partner/spouse (n = 250)

**Factor**	**Total**	**Disclosed (n = 102) N (%)**	**OR (95% CI)**	**P-value**
***Occupation status:***				
Not employed	197 (78.8)	77 (39.1)		
Employed	53 (21.2)	25 (47.2)	1.4 (0.8-2.6)	0.288
Income status (TZS)*:				
≤ 100,000/=	207 (82.8)	77 (37.2)		
> 100,000/=	43 (17.2)	25 (58.1)	2.3 (1.2-4.6)	0.011
***Accommodation:***				
Own house/rent house	133 (53.2)	60 (45.1)		
Renting a room	117 (46.8)	42 (35.9)	0.7 (0.4-1.1)	0.088
***Number of household members***				
≤ 4	171 (68.4)	76 (44.4)		
> 4	79 (31.6)	26 (32.9)	0.6 (0.4-1.1)	0.085
***Number of children <18 years in the household***				
≤ 2	138 (55.2)	35 (38.4)		
> 2	53 (44.8)	19 (30.2)	0.7 (0.4-1.3)	0.258
***Dependent on partner for food/rent/fees***				
No	207 (82.8)	74 (35.7)		
Yes	43 (17.2)	28 (65.1)	3.4 (1.7-6.7)	<0.001

*Exchange rate: **1 **USD = **1490 **TZS in 2010.

**Table 3:** Relationship between sexual and reproductive characteristics and HIV serostatus disclosure to partners (n = 250)

**Variable**	**Total N (%)**	**Disclosed (n = 102) N (%)**	**OR (95% CI)**	**p-value**
***Gravida***				
Multiparous	205 (82.0)	72 (35.1)		
Nuliparous	45 (18.0)	30 (66.7)	3.7 (1.9-2.3)	<0.001
***Living children***				
≥ 3	53 (21.2)	16 (30.1)		
< 3	197 (78.8)	86 (43.7)	1.8 (0.9-3.4)	0.07
***Time since knowing HIV status***				
≤ 12 months	218 (87.2)	79 (36.2)		
> 12 moths	32 (12.8)	23 (71.9)	4.5 (2.0-10.2)	< 0.001
***When diagnosed with HIV***				
During current pregnancy	208 (83.2)	68 (33.7)		
Before current pregnancy	48 (16.8)	34 (70.8)	4.85 (2.4-9.5)	< 0.001
***Discussed with partner before testing***				
No	213 (85.2)	70 (32.9)		
Yes	37 (14.8)	32 (86.5)	13.1(4.9-35.0)	< 0.001
***Ever received couple counseling***				
Yes	41 (16.4)	41 (100)		
No	209 (83.6)	61 (29.3)	0.29 (0.24-0.36)	< 0.001
***Ever used condoms***				
No	104 (41.6)	31 (29.8)		
Yes	146 (58.4)	71 (48.6)	2.2 (1.3-3.8)	0.003
***Knowledge on MTCT and PMTCT:***				
Adequate	178 (71.2)	76 (42.7)		
Inadequate	72 (28.8)	26 (36.1)	0.8 (0.4-1.3)	0.34

The majority of the women (80.8%) learned of their HIV status during their current pregnancy and the time lapse from knowing their status to interview ranged from 1 to 84 months, (median of 5 months). Few women 15% (37) discussed with their partners about HIV testing before taking the test. Of all 250 women, 16% (39) of the pregnant women’s partners came to the clinics for HIV counseling and testing and 23% (58) knew their partners HIV status.

All women reported to have received counseling on infant feeding options and on availability of antiretroviral drugs (ARVs) to reduce MTCT of HIV. Further, 9 out of 10 women reported to have received counseling on importance of using condoms, on importance of partner disclosure and on bringing partner for HIV counseling and testing, see Table 4.

**Table 4:** Proportion of HIV-positive women counseled on different mother-to-child transmission of HIV topics (n = 250)

**Variable**	**Number (Yes response)**	**%**
**Counselor discussed with you about:**		
Use of ARV to reduce MTCT of HIV	250	100.0
Bringing child for ARV after delivery	240	96.0
Infant feeding options	250	100.0
To make decision on feeding mode before infant is born	232	92.8
Importance of contraceptives after delivery	246	98.4
Importance of condom use during sex with partner	246	98.4
Importance of bring partner for HIV counseling at clinic	242	96.8
On disclosure of your HIV status to partner	245	98.0

### Prevalence of HIV-serostatus disclosure and patterns

The proportion of women who had disclosed their HIV serostatus to their partners/spouses was 41% (102/250). Of the 102 respondents who disclosed their sero-status to partners, 80% did so within 7 days after getting the results, while the rest revealed their sero-status sometime later.

**Figure 1:**
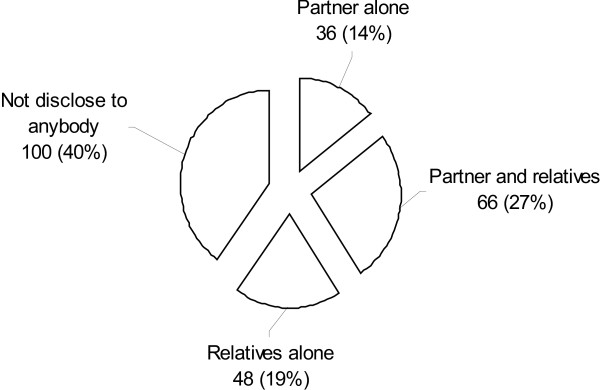
Distribution of respondents according to whom they disclosed their HIV-sero status (n = 250).

In total, 60% (150/250) of the pregnant women had disclosed their HIV status to either partner or other family member at the time of interview, see Figure 1.

Among 148 pregnant women who had not disclosed, 43% (64) planned to disclose to their partners in the future, while 84 women did not plan to disclose at all, to their sexual partners.

### Predictors of HIV self disclosure to partners/spouse

Tables 1, 2 and 3 show the results of bivariate analysis of predictors for HIV sero-status disclosure to partners. Women were more likely to disclose their HIV status to partners if; they were younger than 25 years (p = 0.03), nulliparous (p < 0.001), had secondary or higher education (p = 0.007), had partners who had secondary or higher education (p = 0.008), had high income (p = 0.01) and did not depend on their partner for payment of food/rent/school fees (p < 0.001).

Discussion with partner before testing (OR =13.0), ever used condoms (OR =2.2), and knowledge of woman’s HIV status before current pregnancy (OR = 4.9) positively influenced HIV disclosure to the partner. Awareness of partners HIV status and was associated with HIV disclosure; 98% of pregnant women who knew their partners HIV status disclosed compared to 23% who didn’t, p < 0.001.

Perception of benefit of disclosure and counselors advice also influenced disclosure. Women who perceived there is benefit were 3 fold more likely to disclose compared to those who didn’t perceive there is benefit OR 3.11 (95% CI 1.37-7.09). Also women who perceived counselor did not played a role in disclosure were less likely to inform their partners about their HIV status compared to those who appreciated counselors role OR 0.01 (95% CI 0.00 – 0.02).

Religion, occupation of the women or their partner, partners age, parity, knowledge of PMTCT transmission or prevention were examined but were not associated with HIV sero-status disclosure.

In logistic regression analysis (Table 5), women had increased odds of disclosing their HIV status if; were aged < 25 years (Adjusted odds ratio AOR = 2.2; 95% CI: 1.2-4.1), had discussed with partners before testing (AOR = 6.9; 95% CI: 2.4-20.1 and had knowledge of their HIV status before the current pregnancy (AOR = 3.7; 95% CI: 1.7-8.3). Dependency on partner for food/rent/fees decreased the odds of disclosure by 60%.

**Table 5:** Logistic regression of predictors for self disclosure of HIV-serostatus to partner/spouse among pregnant women (n = 250)

**Variable**	**Disclosed (n = 102) AOR**^**†**^	**95% CI**	**P-value**
***Age of participants(years)***			
≥ 25 years	Reference		
< 25 years	2.2	1.2-4.1	0.01
***Gravida***			
Multiparous	Reference		
Nulliparous	3.3	1.4-7.4	0.005
***Age difference between partners (years)***			
≤ 10 years	Reference		
> 10 years	2.5	1.1-5.6	0.03
***Dependent on partner for food/rent/fees***			
No	Reference		
Yes	0.4	0.1-0.7	0.01
***Discussed with partner before testing***			
No	Reference		
Yes	6.9	2.4-20.12	<0.001
***When diagnosed with HIV***			
During current pregnancy	Reference		
Before current pregnancy	3.7	1.7-8.3	<0.001

^† ^Adjusted for, age education level, income, gravida, duration of living together, time for HIV diagnosis, age difference between male and female, dependency on partner status and discussing with partner before testing and condom use.

## Discussion

This study shows that a relatively low proportion (41%) of mostly married/cohabiting HIV positive pregnant women attending antenatal care in Morogoro Municipality had disclosed their HIV sero-status to their partners. This prevalence of HIV sero-status disclosure is within the range of 17% - 46% reported among pregnant women in Tanzania, Uganda, Kenya, Burkina Faso, and Ivory Coast [8,9,14,19,21,25]. The time of disclosure in most of the studies mentioned ranged from 2 months to 18 months after testing.

There is improvement of HIV disclosure to partners among pregnant women in Tanzania from 17 -22% in 2001 to 41% in 2010 in the current study [9,25]. However this proportion is relatively small given the short period of time before birth where pregnant woman has to make choices on infant feeding options, prolonged ARV use for both herself and the infant, and condom use, all which need partners support [4-6,13,14] and also given the high prevalence of HIV sero-discordance among stable partnerships in Africa including Tanzania [17,18,20]. If the aim is to eliminate HIV transmission to children by 2015, more efforts on sensitization of the community at large to the benefits of HIV disclosure and couple testing are required so that women will be able to adhere to the cascade of PMTCT interventions and prevention of HIV sexual transmission [2].

Findings from this study showed that there is an association between financial dependency and HIV sero-status disclosure to partners. Participants who depended on their partners for food, house rent, and school fees were less likely to disclose their HIV-serostatus to their partner/spouse, than others. Other markers of low economic status such as low income and lower educational level of the woman or her partner also affected disclosure negatively in bivariate analysis. Previous studies in Tanzania and South Africa showed that women with low social economic status or financial dependence on their partner tend to disclose to relatives or others rather than their own partner [25,26]. Fear of loss of economic support by the woman is a reality and a major issue in this set-up where 77% of women had low education, 79% were not employed and 83% had no income or earned less than the minimum wage of 100,000 Tanzanian shillings (~ 67 USD) per month according to 2010 exchange rates. Lack of economic power and dependency to partners not only affect disclosure but have been associated with poor indicators of maternal and neonate health such as low levels of skilled birth attendance at delivery, low attendance for postnatal care, earlier age at sexual debut and high HIV prevalence [27,29-31].

Women who had discussed HIV testing with their partners before undertaking the HIV test were seven times more likely to disclose to their partner than those who didn’t [6,26,32]. In this study, living with a spouse/partner did not influence disclosure as also observed by Antelaman et al., in Dar es Salaam and Makin et al. in South Africa [25,26]. However it was matters related to couple communication which was positively associated with HIV sero-status disclosure; for example condom use which needs agreement between partners, and awareness of partners HIV status. A similar association between partner disclosure and issues related to condom use and communication were observed in Ethiopia, Uganda and South Africa [23,24,26,32]. Communication between partners is vital in enabling a couple to engage in HIV sexual and perinatal prevention. Couple counseling might be one way of helping couples to start communication on HIV and PMTCT issues and needs to be strengthened, as only 16% of the partners in this study came for counseling and testing [11,12].

Time of HIV diagnosis significantly influenced HIV disclosure to partners. Women who knew their HIV status for more than 12 months or knew their status before pregnancy disclosed their HIV status more than others. It might be due to the fact that they had enough time to think and accept their status, therefore finding it easier to disclose, than those who were recently diagnosed and were still in shock and struggling with the HIV diagnosis [22]. Disclosure needs time, and other researchers showed that, rates of HIV sero-status disclosure to partners are much higher among women tested in VCT or CTC centers compared to women tested during pregnancy [7,11,22]. There is a need to shift emphasis and empower women to know their HIV status before pregnancy. Provider initiated counseling and testing (PITC) needs to be strengthened in these facilities as it will increase the number of reproductive age women who know their HIV status earlier [3].

Our results also point to missed opportunities by the health system of diagnosing women of reproductive age earlier for HIV. Most of the participants were multiparous 82% (202/250), and among them 98% had children ≥ to 2 years, (median age of children 3 years, IQR 2–4 years). These women had multiple contacts with health facilities when they brought their children for vaccination, child growth monitoring or when they attended for family planning services. The need to integrate or strengthen PITC for HIV at reproductive and child health (RCH) service delivery points other than antenatal cannot be over emphasized, given that multiparous women in this study were significantly less likely to disclose compared to nulliparous women.

This study had some limitations. Disclosure of HIV status is a sensitive topic among HIV positive women [7,11,26]. These results depended on each woman’s self report and was not verified by their partner. We cannot rule out social desirability bias where women reported what they think the society expects. Secondly, the period of disclosure of HIV sero-status which was reported for this work was limited to the duration of the pregnancy, so our prevalence estimate may be lower compared to studies which followed women for sometime after delivery and we could not assess factors that may influence decision to disclose later such as new partnership or decision to have other children [9,25,26]. Lastly, because of the cross sectional nature of the study, we cannot document whether those who indicated that they have intended to disclose, actually did so and we also did not address the outcomes of disclosure. The strength of the study lies in the high antenatal attendance (98%) by pregnant women in the region, and with the high study response rate. The results of this study may be representative of pregnant women in the area.

## Conclusion

In conclusion, disclosure of HIV sero-status has been identified as an important strategy to reduce vertical and sexual HIV transmission in Tanzania [3,5,29]. While there is an increase in HIV sero-status disclosure to partners among pregnant women during pregnancy in Tanzania (17% to 41%), still 6 out of 10 women enrolled in the study didn’t disclose to their partners. Factors pertaining to discussion/communication between partners, economic dependency, duration of knowledge of HIV status and age influenced HIV sero-status disclosure.

It is recommended that counselors should be encouraged to help women to develop an individualized disclosure plan especially targeting older women ≥ 25 years, multipara women and those who didn’t discuss with partners before testing. Strengthening and integration of PITC into other RCH services is recommended. Couple counseling and broader measures addressing women’s economic empowerment should be long term goals at improving HIV disclosure and partner involvement.

## Competing interests

The authors declare that they have no competing interests.

## Authors’ contributions

ESK designed the study, collected the data, analysis, interpretation and drafted the manuscript. DJD advised on the design, participated in data analysis, interpretation and critical review of the manuscript. SEM provided advise on the design, data collection, interpretation and critical review of the manuscript. All the authors read and approved the final draft of the manuscript.

## Pre-publication history

The pre-publication history for this paper can be accessed here:

http://www.biomedcentral.com/1471-2458/13/433/prepub

## Supplementary Material

Additional file 1 Questionnaires. Click here for file
