# Supplementary material for: Predictors of HIV serostatus disclosure to partners among HIV-positive pregnant women in Morogoro, Tanzania
Source: BMC Public Health. 2013 May 3;13:433. doi: 10.1186/1471-2458-13-433 (PMC3668140; doi:10.1186/1471-2458-13-433)
Supplement: Additional file 1 — Questionnaires. [file 1471-2458-13-433-S1.pdf]

APPENDIX 2: Questionneres

Client no--.

Name of the clinic/ hospital------------------ Ward……………

Gestation age:…... Gestation age when start ANC care…………..

No of times the woman has attended for ANC care……………………………………

S.N	QUESTION	OPTIONS	CODE NO.
**PART I- Demographic and household information. (Objective 2)**			
1	When were you born?	Month/year	( )
2.	What is your religion?	01Christian<br>02 Muslim<br>03 Non region<br>03Other specify	( )
3	Are you employed and receive regular monthly salary?	01Yes. → 4<br>02 No. → 5	( )
4	What is you present occupation?	---	
5	If you are not employed, what do you do to earn income?	01 Farmer<br>02 Peasant<br>03 Business<br>04 Other specify	
6	What is the highest level of education you have attained?	01 No formal education<br>02 Primary education (class…….)<br>03 O level education (Form……)<br>04 A level education<br>05Highereducation Diploma or degree	( )
7	What is your approximate income per month?	01 None depend on husband/ relatives /parents<br>02 $\leq$ 30,000 Tsh per month	( )

		(≤ 30 USD per month)<br>03 > 30,000 -100,000 Tsh per month<br>04 > 100,000 – 200,000 Tsh per month+<br>05 > 200,000 Tsh per month	
8	What is your current marital status?	01 Married → 10<br>02 Cohabiting → 10<br>03 Single<br>04 Separated (for how long………)<br>05 Divorce (for how long………)<br>06 Widow (for how long……)	( )<br><br>--------------
9	What was your marital status when you learned you were HIV infected?	01 Married<br>02 Cohabiting<br>03 Single<br>04 Separated<br>05 Divorced<br>06 Widow	( )
10	For those married/ cohabiting how long have you lived together?-----------------		
11	Are you in polygamous relationship?	01 Yes<br>02 No	( )
12	For the singles, separated, divorced how long have you known or lived with the man who is a father of your current pregnancy------------------------------------		
13	How do you live/ living style?	01. Living together all the time<br>02. Visits<br>03. Living apart > 6 months	(}
14	What is your partners' age?--		

15	What is his level of education?--		
16	What is your partners' occupation?.------------------------------------		
	**Household information**		
17	What is the total number of people in your household?<br>How many are children, i.e. below 18 years……………………………		
18	The house you and your family lives, is it your own house or you are renting?	01. Our own house<br>02. Renting a whole house<br>03. Rent a room<br>04.Others…………………<br>………..	( )
19	Who usually pay for the following things at your house?<br><br>Myself My partner Share the costs<br>Purchase food ……… ……… ………<br>Pay for the house rent ……… ……… ………<br>Pay for the school fees ……… ……… ………		
	**Reproductive health**		
20	Gravida number…………………..<br>*For gravida 2 or more (fill Q 21-25)*		
21	How many children did she give birth…………………..		
22	How many living children does the woman has/have?..........................		

23	**History of:**<br>Miscarriage/abortions<br>Child born alive and died < & year	Yes<br>…..<br>…..	No<br>…..<br>…..
24	24. Age of the last born ………………………………………………………		
25	Do all your children have the same father	01. Yes ( )<br>02. No	
25	25. Do all your children have the same father?	01 Yes<br>02 No	( )
26	26. Have you ever used any family planning method before this pregnancy?	01 Yes<br>02 No	( )
27	Have you ever used condoms	01 Yes<br>02 No	( )
28	Currently, are you using condoms when you have sex with your partner?	01 Yes<br>02 No	( )
	**PART III. Knowledge of PMTCT**		
29	Can HIV infected woman transmit the infection to her baby?	01. Yes<br>02. No<br>03. Don't know	( )
30	If YES to question **29**, when or at which time can the transmission occur? **(Tick all the possible answers the woman mention**)	01 During pregnancy------<br>02 During labour and delivery------<br>03 During breastfeeding period-----<br>04 Others:……………………	

31	Is it possible for HIV positive pregnant mother to prevent her baby from been infected?	01 Yes<br>02 No<br>03. Don't know	( )
32	How can a pregnant HIV positive mother prevents her baby from getting HIV+ infected? (multiple answers are possible)	01. By taking antiretroviral prophylaxis during pregnancy<br>02. Caesarian section<br>03. Exclusive breastfeeding<br>04. Exclusive replacement feeding (AFASS)<br>05.Others……………………	( )
	**PART IV. Disclosure patterns**		
33	When were you first diagnosed HIV positive?	Month/year--------------------------------------	
34	Did the woman discover she is HIV positive during current pregnancy or before?	01. Knew during the current pregnancy<br>02. Knew before this pregnancy	( )
35	Did you discuss with your partner about HIV testing, before you took the test?	01. Yes<br><br>02. No	( )
36	Have you ever disclosed your serostatus?	01. Yes<br><br>02. No --- 42	( )
37	Have you disclosed your serostatus to your partner?<br>Have you told your husband/spouse or partner that you are HIV positive	01 Yes → 38 – 41<br>02 No → 42, 43	( )
38	How long after getting your results did you tell your partner you are HIV positive?	01. Same day<br>02. Others (specify----------------------------	

39	What was his reaction when you disclosed?	01. He did nothing<br>02. Positive reaction (specify----------------<br>03. Negative reaction (specify---------------	
40	Relationship with your partner before testing and disclosure to him.	01. Smooth relation<br>02. With disagreement	( )
41	Relationship with your partner AFTER disclosing your results	01 Smooth relation<br>02With disagreement	( )
42	**If no;**<br>Are you planning to disclose to him before delivery?	01. Yes<br>02. No	( )
43	What do you think will be the reaction of your partner IF you tell him you are HIV positive?	01 He will do nothing<br>02 Anticipating a positive reaction (specify…………..<br>03 Anticipating a negative reaction (specify	( )
44	Who else did you disclosed your serostaus to?	01. Nobody<br>02. Parents.<br>03 Friends<br>04 Sister<br>05 Brother<br>06 Other specify	( )
45	Has your partner come for HIV counseling and testing, here at the clinic/hospital?	01. Yes<br>02. No	( )
46	Do you know his HIV status	01. Yes<br>02. No	( )
47	Ever received couple counseling with your current partner?	01. Yes<br>02. No	( )
48	Do you think the counselor played a role in influencing your decision on self disclosure to your partner?	01. Yes<br>02. No	( )

	**PART V. Knowledge on advantages of self disclosure to partners, perceived benefits & barriers.**		
49	Do you think there are any benefits of disclosing your HIV sero status to your partner?	01 Yes → 50<br>02 No → 51	( )
50	If Yes what are the benefits of disclosing your HIV serostatus to partner?	01 ---------------------------.------------------<br>02 --<br>03--<br>04--	
51	. If no, why not?	01 --<br>02--<br>03--.<br>04 --	
52	**Do you think** self disclosure among HIV positive pregnant women to partners increases ARV prophylaxis uptake among women	01 Yes<br>02 No	( )
53	**Do you think** self disclosure to partners helps in ARV adherence to pregnant women	01Yes<br>02 No	( )
54	**Do you think** self disclosure of HIV status to partner helps HIV positive pregnant women to bring/ give ARV prophylaxis to their infants after delivery?	01Yes<br>02 No	( )
55	**Do you think** self disclosure among HIV positive pregnant women to their partners helps them to *choose* the infant feeding method they want?	01 Yes<br>02 No	( )
56	**Do you think** self disclosure among HIV positive pregnant women to their partners helps them to *adhere* to the infant feeding method they will chose/ they have chosen	01Yes<br>02 No	( )

57	**Do you think** Self disclosure to partners among HIV positive pregnant women helps in future plans for further pregnancies	01Yes<br>02 No	( )
58	**Do you think** self disclosure to partners among HIV positive pregnant women helps in discussions and future use of family planning	01Yes<br>02 No	( )
59	**Do you think** self disclosure to partners among HIV positive pregnant women helps in discussion and future use of condoms	01Yes<br>02 No	( )
60	Does self HIV disclosure to partners prevent your partner from getting the infection if he is negative	01Yes How?-----------------<br>02 No ( )	
61	Does self HIV disclosure to partners prevents a baby from getting the HIV infection?	01 Yes How?-----------------<br>02 No	
62	64. Does self HIV disclosure to your partner benefit you yourself?	01 Yes How-------------------<br>02 No ( )	
63	What can be the effects if HIV positive pregnant mother did not disclosed her serostatus to her partner?	01 ---<br>02---<br>03---<br>04---	
64	In your area, what are the barriers most HIV pregnant women have in disclosing their status to their husbands/partners?	01 ---<br>02---<br>03---<br>04---	
65	In your area, what are the barriers most HIV pregnant women have in disclosing their status to their family members apart from partners?	01 ---<br>02---<br>03---<br>04---	

PART V Attitudes of HIV positive pregnant women towards self disclosure to partners

66	It is good for pregnant women to share their serological results to their partners as early as possible	01Strongly agree.<br>02Agree<br>03Strongly disagree<br>04 Disagree	( )
67	Discussion among partners before test it helps pregnant women to disclose their serostatus to their partners	01Strongly agree.<br>02Agree<br>03Strongly disagree<br>04Disagree	( )
68	Married pregnant women they found easily to disclose their serostatus.	01Strongly agree.<br>02Agree<br>03Strongly disagree<br>04Disagree	( )

69. During the post test counseling session, did the counselor discuss with you about the following issues/ topics? ***Tick where appropriate***

	Yes	No
a) You, taking ARV to reduce HIV transmission to the child	....	....
b) Bringing the child after delivery to get ARV to reduce MTCT of HIV	....	....
c) Infant feeding methods	....	....
d) Discussed until you choose infant feeding method you will use	....	....
e) Discussed about importance of using family planning after delivery	....	....
f) Importance of using condoms during sex with your partner	....	....
g) Importance of involving or bring your partner for testing	....	....
h) Importance of disclosing your serostatus to your partner?	....	....
i) Discussed and made a personal plan on how you should disclose to partner	---	----
